# Psychological predictors of risky driving: the role of age, gender, personality traits (Zuckerman’s and Gray’s models), and decision-making styles

**DOI:** 10.3389/fpsyg.2023.1058927

**Published:** 2023-05-18

**Authors:** Anton Aluja, Ferran Balada, Oscar García, Luis F. García

**Affiliations:** ^1^Deparment of Psychology, University of Lleida, Lleida, Spain; ^2^Lleida Institute for Biomedical Research, Dr. Pifarré Foundation, Lleida, Spain; ^3^Department of Psychobiology and Methodology of Health Sciences, Autonomous University of Barcelona, Catalonia, Spain; ^4^Deparment of Psychology, European University of Madrid, Madrid, Spain; ^5^Deparment of Biological Psychology and Health, Autonomous University of Madrid, Madrid, Spain

**Keywords:** personality traits, decision-making styles, Zuckerman’s alternative five factor personality model, Gray’s personality model, risky driving

## Abstract

The present study was planned to study the relationships between age, personality (according to Zuckerman’s and Gray’s psychobiological models) and decision-making styles in relation to risky driving behaviors. The participants were habitual drivers, 538 (54.3%) men and 453 (45.7%) women, with a mean age around 45 years and mainly of middle socioeconomic status. The results indicate that the youngest men and women reported more Lapses, Ordinary violations, and Aggressive violations than the oldest men and women. Women reported more Lapses (*d* = −0.40), and men more Ordinary (*d* = 0.33) and Aggressive violations (*d* = 0.28) when driving. Linear and non-linear analysis clearly support the role of both personality traits and decision-making styles in risky driving behaviors. Aggressiveness, Sensitivity to Reward, Sensation Seeking played the main role from personality traits, and Spontaneous and Rational decision-making style also accounted for some variance regarding risky driving behaviors. This pattern was broadly replicated in both genders. The discussion section analyses congruencies with previous literature and makes recommendations on the grounds of observed results.

## 1. Introduction

Victims and injuries from traffic accidents are considered a serious social and health problem. A global status report on road safety by World Health Organization (2018) showed that road crashes are the leading cause of death for young adults under 29 years of age (Zeyin et al., 2022). In 2021, there were 1,004 deaths and 3,728 serious injuries in traffic accidents in Spain.^1^ Among the most common human causes of traffic accidents are distractions such as the use of smartphones, fatigue, high speed, alcohol or other drugs, lack of experience, or drowsiness (Bucsuházy et al., 2020). Dangerous driving is a serious traffic offense in which the driver of a vehicle does not respect traffic regulations, and it is responsible for many traffic violations and accidents causing deaths and other damage. An aggressive/risky driving behavior refers to habitual driving behavior dominated by excitatory motives (Sagberg et al., 2015).

There are many questionnaires to assess risky or aberrant driving behavior. The Manchester Driver Behavior Questionnaire (DBQ; Reason et al., 1990) is well known, and it has a robust factorial structure of four factors: *Lapses, Errors, Ordinary violations, and Aggressive violations* (Lajunen et al., 2004). Lapses are considered attention and memory problems, such as forgetting where your car was parked. Errors are failures in observation or misjudgments, such as braking too quickly. Ordinary violations are behaviors such as speeding or driving too close to another vehicle. Aggressive violations are related to the expression of hostility toward other drivers or making rude gestures (Gras et al., 2006). It is important to remark that lapses and errors are mainly unintentional, whereas violations, especially aggressive violations, are intentional and sufficiently strong to override the perceived risks related to committing the violation. In a meta-analysis, de Winter and Dodou (2010) reported that violations were predictors of traffic accidents.

Risky driving has been linked to several factors such as age, gender, and individual differences dimensions (Hassan and Abdel-Aty, 2013), certain disinhibited personality traits [for example, adventure-seeking traits that lead to an underestimation of risk (Jonah, 1997; Zhang et al., 2019)]. Moreover, driving is a multifaceted decision-making process. Errors at different stages of these processes may lead to situations of risk and contribute to accidents (Gugliotta et al., 2017). Therefore, decision-making style could be an individual variable to consider in motor driving risk research.

### 1.1. Age, gender, and driving

Young drivers are riskier and have higher mortality (Tefft, 2017; Alderman et al., 2018; Das et al., 2018). This association between age and risky driving has been reported in both Western and Eastern countries (McCartt et al., 2009; Zeyin et al., 2022). Furthermore, young men perceive themselves as more self-confident, although they are more distractible and engage in more risky behavior compared to women (Barr et al., 2015). In this sense, age has also been negatively related to Lapses, Errors, Ordinary violations and Aggressive violations using the DBQ (Berdoulat et al., 2013; Lucidi et al., 2019). The effect of age on driving also differs according to gender. For instance, comparing male and female teenagers, adolescent male had more excess speeding crashes than female (Swedler et al., 2012).

Gender differences have also been noted, with women scoring more on Lapses, and men presenting more Ordinary violations and Aggressive violations (Lajunen et al., 2022). A recent study examines the relationship between road rage and masculinity-femininity gender roles in young drivers (Deniz et al., 2021). The results indicated that masculinity and anger were positively related to impolite behaviors in men, but femininity was negatively related to verbal aggression while driving. According to the authors, these results suggest that gender moderates the relationship between road rage among young drivers. Some gender differences in driving style could be related to gender differences in personality, including Sensation Seeking, Aggressiveness and impulsivity traits (Cross et al., 2011, 2013).

### 1.2. Personality traits and driving

Since the 1970s certain disinhibited personality traits, such as Sensation Seeking, have been studied in relation to risky driving and speeding (Zuckerman and Neeb, 1980). The Sensation Seeking trait proposed by Zuckerman is defined as “*The seeking of varied, novel, complex, and intense sensations and experiences and the willingness to take physical, social, legal, and financial risks for the sake of such experiences*” (Zuckerman, 1994, p. 27). It is a trait associated with social and antisocial risk behaviors of different types, including risky driving (Jonah, 1997; Jonah et al., 2001; Lucidi et al., 2010, 2019). The most recent meta-analysis is that of Zhang et al. (2019), which showed significant correlations of Sensation Seeking with risky driving, aggressive driving, errors, and accident involvement. Most studies used the Sensation Seeking Scale, Form V (SSS-V; Zuckerman et al., 1964), but there is a new version within Zuckerman’s questionnaire of five factors, the Zuckerman-Kuhlman-Aluja Personality Questionnaire (ZKA-PQ; Aluja et al., 2010), which includes a revised Sensation Seeking dimension with four facets (see the review of Zuckerman and Aluja, 2015): Thrill and adventure seeking, Experience-seeking, Disinhibition and Boredom susceptibility/impulsivity.

Aggressiveness, as a personality trait, has also been linked to risky driving style. Iancu et al. (2016) carried out a meta-analysis about the association between personality and aggressive driving. They compared two personality models: The Five Factor Model (FFM) and Zuckerman’s Alternative Five model (AFFM). Regarding FFM, the results indicated a significant association between Neuroticism and Agreeableness with Aggressive driving, and a marginal one with Extraversion. Regarding the AFFM (using the old ZKPQ; Zuckerman et al., 1993), a significant effect was also found for Anxiety-Neuroticism, a marginal effect for Sociability, and a moderate effect for Aggression-hostility. It should be noted that the AFFM was revealed to be more related to aggressive driving than FFM (Iancu et al., 2016). A driving study used the ZKA-PQ (Martí-Belda et al., 2019) in which the authors compare the scores on the ZKA-PQ in three groups of drivers: Group A Non-offenders, Group B Court Offenders and C Penalty Point Offenders. Groups B and C showed significantly higher scores than Group A in the Sensation Seeking dimension. Furthermore, these two groups also scored significantly higher in Aggressiveness, Activity, and Neuroticism (Martí-Belda et al., 2019). It is worth noting that Sensation Seeking has been related to Hostility and Aggressiveness (Aluja and Torrubia, 2004).

Anger at the wheel is also related to aggressive driving and this would be associated with impatience, annoyance, hostility and/or an attempt to save time (Tasca, 2000). Demir et al. (2016) performed a meta-analysis on driving anger and found that anger (physical and verbal aggression expressions) was significantly associated with the behavior of violations and errors. Drivers high on the anger rating tend to perceive many other drivers’ behavior as intentionally aggressive (Kerwin and Bushman, 2020). Verbal aggressiveness has been linked to self-reported driver aggression, while physical aggressiveness has been linked to aggressive behavior (Lajunen and Parker, 2001). In another study, aggressiveness was a robust predictor for risky driving for young men, but not for women (Šeibokaitė et al., 2014). Related with anger, the more general Neuroticism trait has been linked to errors and accidents when driving (Alavi et al., 2017). In this way, Wang et al. (2018) found that Neuroticism was the best personality predictor of errors and lapses. People with high neuroticism trait are at greater risk of errors and lapses when driving because they are more easily distracted while driving (see also Hansen, 1989). From Gray’s Reinforcement Sensitivity Theory (RST), Neuroticism is represented by the Sensitivity to Punishment trait (Corr, 2004; Aluja and Blanch, 2011).

On the other hand, the inhibition deficits related to impulsivity can lead to aggressive behavior behind the wheel (Čabarkapa et al., 2018). In addition, Impulsiveness and Sensation Seeking predict behaviors related to crashes, aggressive driving, risky driving, and expression of anger while driving (Dahlen et al., 2005). Gray’s Reinforcement Sensitivity Theory (RST) has been used extensively in research on impulsivity and driving (Castellà and Pérez, 2004; Scott-Parker et al., 2012, 2013; Mohammadzadeh-Ebrahimi and Alavi, 2021). Scott-Parker and Weston (2017), in a revision of literature about Sensitivity to Reward and risky driving, concluded that young drivers with higher Sensitivity to Reward drive in a riskier way, drive faster, have more crashes, and have more violations. Sensitivity to Reward is also associated with Sensation Seeking (Aluja et al., 2013), and both traits are related to risky driving, mostly in young men (Scott-Parker et al., 2013). The literature review reports that men are more impulsive than women (Cross et al., 2011), and take part in riskier behaviors when driving.

### 1.3. Decision making style and driving

Decision-making style is a cognitive variable since it depends on how people process information (Hunt et al., 1989; Urieta et al., 2022). One of the most used decision-making instruments in research is the General Decision-Making Styles (GDMS) proposed by Scott and Bruce (1995). The GDMS includes five decision-making styles: *Rational, Intuitive, Dependent, Avoidant, and Spontaneous*. *Rational* decision-making style involves the use of reasoning, logical and structured approaches to decision-making. *Intuitive* decision-making style is defined by reliance upon hunches, feelings, impressions, instinct, and good feelings. *Dependent* style is defined by a search for advice and guidance from others before making important decisions. *Avoidant* decision- making style is defined by withdrawing, postponing, moving back and negating the decision scenarios. A *Spontaneous* style is characterized by a feeling of immediacy and a desire to get through the decision-making process as quickly as possible (Scott and Bruce, 1995). Women have higher average scores in the Dependent and Intuitive styles, and men in the Rational one (Chen, 2013; Delaney et al., 2015; Bayram and Aydemir, 2017). Men also have higher average scores in the Spontaneous one (Alacreu-Crespo et al., 2019).

Previous studies report that drivers with a Rational decision-making style seek relevant information, assess the consequences and act logically, while Intuitive drivers do not anticipate the consequences of their actions when making their decisions. So, the lower the score in the Rational style, the greater the possibility of taking risks while driving. There were also positive associations between risky driving behaviors and Spontaneous and Avoidant decision-making styles. In general, decision-making style is related to driving and certain styles can increment the likelihood of having a traffic accident (French et al., 1993; Barati et al., 2020).

### 1.4. Aims of the present study

As far as we know, there are many studies analyzing the role of age, gender, personality traits and decision-making styles in risky behavior, but none have considered all these variables altogether. Hence, the general aim of this study is to examine at the same time and in a large sample of drivers the role of age, gender, personality -according to Zuckerman’s and Gray’s models- and decision-making style in risky driving behavior to obtain a description of the risky driver. According to the studies reviewed, the following things are expected: (a) a negative association between age and risky driving, (b) significant higher scores of women in driving lapses, while men would have higher scores in ordinary/aggressive violations, (c) Aggressiveness, Sensation Seeking and Reward to Sensitivity would be related to some aspects of risky driving, and (d) people with Rational decision-making styles are expected to have fewer Lapses, Errors, and Ordinary/aggressive violations, while avoiders and, especially, spontaneous people would have more Lapses, Errors, and Ordinary/aggressive violations. We also compare the predictive power of personality traits and decision-making styles regarding risky driving.

## 2. Method

### 2.1. Participants and procedure

The sample consisted of 991 participants, who were habitual drivers of motor vehicles with 2 or 4 wheels. 538 (54.3%) were men and 453 (45.7) were women. The mean ages for men and women were 45.67 (*SD* = 16.07) and 44.04 (*SD* = 15.25), respectively. The age range was 18–90 years with a normal distribution (kurtosis = −0.64 and skewness = −0.14). The sample was obtained from the general population with the help of students, who administered the questionnaire protocol anonymously and voluntarily to healthy members of the community. Each student was required to recruit men and women from the following age ranges: 18–35, 36–45, 46–60, and more than 60 years. The Hollingshead Social Position Index (Hollingshead, 1957; Hollingshead and Redlich, 1958) was obtained using the following formula: (Occupation score * 7) + (Education score * 4). The range of scores 11–17, 18–31, 32–47, 48–63, and 64–77 correspond to upper, upper-middle, middle, lower-middle, and lower social position, respectively. The participants were asked if they had a driving license and if they drove regularly. Those who answered no to either of the two questions were excluded from the study. No information about the type of vehicle used was collected. Participants completed Spanish versions of all questionnaires. The protocol was part of a wider study that included different electrophysiological tests in our Human Behavior Laboratory within the framework of a National Research Project. All participants who performed the laboratory tests signed an informed consent. The study was conducted in accordance with the Declaration of Helsinki and was approved by the ethical commission of the University.

### 2.2. Instruments

#### 2.2.1. Zuckerman-Kuhlman-Aluja personality questionnaire shortened form

The ZKA-PQ/SF (Aluja et al., 2018) is a shortened version of 80 items from the original 200-item ZKA-PQ. The response format is a 4-point Likert-type scale ranging from 1 (*strongly disagree*) to 4 (*strongly agree*). The questionnaire has five personality domains: Aggressiveness (AG), Activity (AC), Extraversion (EX), Neuroticism (NE), and Sensation Seeking (SS) and 20 facets (for details see, Aluja et al., 2018). Note that no analysis was conducted at the facet-level in the present study. The ZKA-PQ and ZKAPQ/SF showed good validity and reliability in the original studies (Aluja et al., 2010, 2018). Both questionnaires have also been validated in numerous cultures and languages (Rossier et al., 2016; Aluja et al., 2022). The ZKA-PQ/SF items are included in the appendix of Aluja et al. (2018).

#### 2.2.2. The sensitivity to punishment and sensitivity to reward questionnaire

This is a 20-item shortened version (Aluja and Blanch, 2011) of the Sensitivity to Punishment and Sensitivity to Reward Questionnaire developed by Torrubia et al. (2001). The SPSRQ-20 has 20 items, 10 items for each scale: Sensitivity to Punishment (SP) and Sensitivity to Reward (SR). SP is considered a measure of anxiety, and SR a measure of impulsivity within Gray’s theory. This instrument has a 4-option Likert type answer format. The SPSRQ 20-item version shows a robust factor structure and a satisfactory adjustment to observed data in a covariance structure context, supporting the use of the shorter version to assess Gray’s Reinforcement Sensitivity Theory (Torrubia et al., 2001). Original alpha internal consistency values for SP were 0.77 (men) and 0.80 (women), and for SR 0.73 (men) and 0.70 (women).

#### 2.2.3. General decision-making scale

The GDMS (Scott and Bruce, 1995) is a self-administered 22-item questionnaire adapted to Spanish by Alacreu-Crespo et al. (2019). The response format is a 5-point Likert-type scale ranging from 1 (*strongly disagree*) to 5 (*strongly agree*). The confirmatory factor analysis supported the five-factor structure of GDMS as well as measurement invariance across gender. Alpha internal consistency values ranged from 0.72 to 0.91.

The GDMS has five different scales, each representing a decision-making style: Rational (RA), Intuitive (IN), Dependent (DE), Avoidant (AV), and Spontaneous (SPO).

#### 2.2.4. The manchester driver behavior questionnaire

The DBQ (Reason et al., 1990) has been used in many studies about risky driving. It has 34 items and was adapted and validated in Spain by López de Cózar et al. (2004, 2005). It has a response format from 0 (never) to 10 (always). The Spanish validation replicated the four-factor structure: Lapses (LA), Errors (ER), Ordinary violations (OVI) and Aggressive violations (AVI). Violations require explanation in terms of social and motivational factors, whereas errors (slips, lapses, and mistakes) may be accounted for by the information-processing characteristics of the individual (Reason et al., 1990).

### 2.3. Analysis of data

Descriptive analysis, and comparisons between genders for age, social position and psychometric variables was performed. Cronbach’s alpha internal consistency value of the scales was also calculated. Graphical comparisons of the DBQ by age and gender ranges were also presented using ANOVA and Scheffe post-test. Additionally, an ANOVA 2×5 (gender x age ranges) was computed for the four scales of the DBQ. Zero-order correlations, independently for men and women, were also considered for all variables. In addition, empirical network analysis was used to analyze the unique relationships between age, ZKA-PQ, SPSRQ-20, GDMS and DBQ scales (GLASSO algorithm, EBIC, and mgm, the last for estimating explained variance; Chen and Chen, 2008; Friedman et al., 2014). Furthermore, both sets of domains were also analyzed together to test the connections between them. The use of this technique makes it possible to estimate the partial correlations between each pair of scales while controlling the inflation of Type I error thanks to the GLASSO regularization technique (Tibshirani, 1996; Epskamp, 2016).

To compute the association and the predictive power of personality and decision-making styles variables with risky driving, two analyses were performed. First, the factorial convergence of all the psychometric scales used was analyzed. In this way, a principal component analysis with Varimax rotation was computed. This analysis allows for testing all associated variables together, and which of them load on the same factor suggesting common variance. Later, a multiple linear regression analysis was performed for men and women separately, using the total DBQ score as dependent variable and age, ZKA-PQ/SF, SPSRQ-20 and GDMS scales as independent variables. Stepwise method was used with a probability-to-enter (PIN) of *p* < 0.05. Every DBQ scale was then predicted after age, personality variables and decision-making styles. The regression was also computed separately for men and women but in this case, the introduction method was used in the regression to allow for comparing all variables across the four DBQ scales.

Nonparametric local LOESS graphical analysis (Fox, 2000) was also performed to detect any nonlinear patterns. The local area nonparametric LOESS polynomial regression procedure was used to produce data points for the fully running DBQ (T-scores) and GDMS and ZKA-PQ/SF-SPSRQ-20 domains (z-scores). The method involves a series of local regression analyses that allows the shape of a curve to vary through the continuous variable. The procedure is a robust adjustment method that is flexible and ideal for potentially revealing complex and unforeseen association patterns between variables (O’Connor, 2005). To conduct this analysis, a global score on DBQ was computed by summing the four scales. This total score can be computed in this way since all DBQ scales present high intercorrelations among them (Table 1). Statistical analyzes have been carried out using the SPSS 26.0 (IBM Corp, 2019) the corrplot (Wei and Simko, 2017) and qgraph (Epskamp et al., 2012) R packages.

**Table 1:** Inter-correlation matrix for age DBQ, ZKA-PQ/SF, SPSRQ-20, and GDMS.

	Age	Extraversion	Neuroticism	Sensation seeking	Aggressiveness	Activity factor	Sensitivity to reward	Sensitivity to punishment	Rational	Intuitive	Dependent	Avoidant	Spontaneous	Lapses	Errors	Ordinary violations	Aggressive violations
Age		−0.16	−0.03	**−0.36**	−0.11	0.02	−0.10	−0.01	0.11	−0.10	−0.11	−0.03	−0.08	−0.11	−0.10	−0.26	−0.17
Extraversion	−0.10		−0**.34**	0.24	−0.22	0.18	0.11	**−0.42**	0.03	0.24	−0.06	−0.26	−0.01	−0.01	−0.04	0.04	0.03
Neuroticism	−0.11	**−0.38**		−0.01	**0.41**	−0.02	0.18	**0.67**	−0.06	−0.17	**0.37**	**0.41**	0.14	0.22	0.16	0.05	0.09
Sensation seeking	**−0.37**	0.27	0.00		0.20	0.24	**0.35**	−0.19	−0.04	0.17	−0.02	0.01	**0.37**	0.15	0.19	**0.30**	0.20
Aggressiveness	−0.17	−0.16	**0.47**	0.11		0.08	**0.30**	0.17	−0.19	−0.02	0.07	0.19	**0.35**	0.27	0.29	0.26	**0.38**
Activity factor	0.04	0.28	−0.07	0.19	0.07		0.15	−0.09	0.11	0.11	−0.07	−0.04	10	−0.01	−0.01	0.03	0.07
Sensitivity to reward	−0.25	0.15	0.24	**0.33**	0.38	0.26		0.09	−0.01	0.07	0.12	0.19	0.28	0.19	0.21	0.21	0.28
Sensitivity to punishment	0.02	**−0.44**	0.**62**	−0.17	0.18	−0.12	0.10		0.04	−0.22	**0.35**	**0.39**	−0.04	0.11	0.03	−0.07	−0.06
Rational	0.12	0.06	−0.12	−0.11	−0.23	0.16	−0.10	−0.14		0.07	0.12	−0.09	**−0.48**	−0.16	−0.12	−0.14	−0.22
Intuitive	0.04	0.19	0.04	0.21	0.13	0.17	0.19	−0.04	0.11		−0.11	−0.20	0.15	0.01	−0.07	−0.03	0.02
Dependent	0.04	−0.08	**0.34**	−0.11	0.10	0.06	0.11	**0.32**	0.15	0.13		**0.34**	−0.10	0.11	0.06	−0.07	−0.08
Avoidant	−0.10	**−0.33**	**0.50**	−0.07	0.25	−0.17	0.17	**0.49**	−0.24	−0.01	0.**31**		0.16	0.23	0.20	0.09	0.11
Spontaneous	−0.23	0.04	0.24	**0.32**	**0.36**	0.04	**0.34**	0.10	**−0.40**	0.20	0.00	**0.31**		0.14	0.09	0.20	0.25
Lapses	−0.22	−0.11	0.27	0.06	0.26	−0.07	0.19	0.20	−0.16	−0.04	0.07	0.11	18		**0.74**	**0.53**	**0.51**
Errors	−0.18	−0.10	0.24	0.07	0.23	0.01	0.26	0.14	−0.15	−0.03	0.06	0.14	22	**0.67**		**0.68**	**0.62**
Ordinary violations	**−0.40**	0.00	0.13	0.28	0.24	−0.02	0.28	−0.02	−0.11	0.01	0.07	0.09	20	**0.47**	**0.61**		**0.69**
Aggressive violations	**−0.34**	0.02	0.13	0.19	**0.43**	0.08	**0.36**	−0.02	−0.10	0.01	−0.02	0.04	0.23	**0.44**	**0.58**	**0.70**	

Lower left represents results for men. Upper right for women. *r*: 0.09 or higher *p* < 0.05; *r*: 0.12 or higher *p* < 0.01; *r*: 0.15 or higher *p* < 0.001. Correlations equal to or greater than 0.30 are in boldface.

## 3. Results

### 3.1. Descriptive statistics of age, social position index, ZKA-PQ/SF, SCSRQ-20, GDMS, DBQ, and gender comparisons

Table 2 shows the descriptive statistics of the two sociodemographic variables (age and social position), and the psychometric scales for each gender. It also reports alpha coefficients as well as t-test of gender comparisons. There were no differences in age and social position between genders. Both men and women reported a social position index around 31, which corresponds to a “middle” level according to Hollingshead (1957). The higher means in men were in Ordinary violations (*p* < 0.001; *d* = 0.33), Aggressive violations (*p* < 0.001; *d* = 0.28), Sensation Seeking (*p* < 0.001; *d* = 0.32) and Sensitivity to Reward (*p* < 0.001; *d* = 0.23). In contrast, women obtained higher means in Lapses (*p* < 001; *d* = −0.40), Intuitive (*p* < 0.001; *d* = −0.22) and Dependent (*p* < 0.001; *d* = −0.22) decision-making style, and personality traits such as Neuroticism (*p* < 0.001, *d* = −0.44) and Sensitivity to Punishment (*p* < 0.001, *d* = −0.43). Alpha coefficients showed adequate values in all scales.

**Table 2:** Descriptive statistics, gender comparisons and Cronbach’s alpha internal consistency.

	Men (*n* = 539)		Women (*n* = 453)		All				*M*	*SD*	*M*	*SD*	*t-*test	*p*<	*d*	*Alpha*
Age	45.62	16.09	44.04	15.24	1.58	0.115	0.10	–
Social position index	31.95	17.81	31.36	17.69	0.47	0.638	0.03	–
**ZKA-PQ/SF**								
Aggressiveness	32.62	8.99	32.54	8.61	0.14	0.888	0.00	0.88
Activity	41.93	7.13	42.11	7.37	−0.40	0.692	−0.02	0.81
Extraversion	47.68	7.78	49.04	7.54	−2.78	0.006	−0.18	0.85
Neuroticism	32.06	9.06	36.05	9.28	−6.84	0.001	−0.44	0.90
Sensation seeking	37.66	8.62	34.87	8.46	5.12	0.001	0.32	0.84
**SCSRQ-20**								
Sensitivity to reward	21.53	5.56	20.31	5.09	4.53	001	0.23	0.79
Sensitivity to punishment	21.51	5.51	23.91	5.80	−8.37	001	−0.43	0.82
**GDMS**								
Rational	4.06	0.64	3.98	0.63	1.97	0.049	0.12	0.86
Intuitive	3.58	0.88	3.77	0.85	−3.46	0.001	−0.22	0.90
Dependent	3.31	0.83	3.49	0.79	−3.58	0.001	−0.22	0.83
Avoidant	2.20	0.93	2.31	0.91	−1.90	0.053	−0.12	0.92
Spontaneous	2.24	0.86	2.25	0.88	−0.24	0.088	−0.01	0.87
**DBQ**								
Lapses	20.45	12.13	25.85	15.32	−6.20	0.001	−0.40	0.84
Errors	8.55	6.70	8.86	7.61	−0.66	0.507	−0.04	0.81
Ordinary violations	17.27	13.31	13.14	11.62	5.15	0.001	0.33	0.84
Aggressive violations	12.58	11.53	9.59	9.52	4.39	0.001	0.28	0.81

ZKA-PQ/SF: Zuckerman-Kuhlman-Aluja Personality Questionnaire shortened form. SPSRQ-20: Sensitivity to Punishment and Sensitivity to Reward Questionnaire shortened 20-item form. GDMS: General Decision-Making Scale. DBQ: Driving Behavior Questionnaire. Cohen’s *d*: 0.10: very small, 0.20: small, 0.50: medium, 0.80: large, 1.20: very large.

### 3.2. Age ranges and gender comparisons by driving behaviors scales

**Figure 1:**
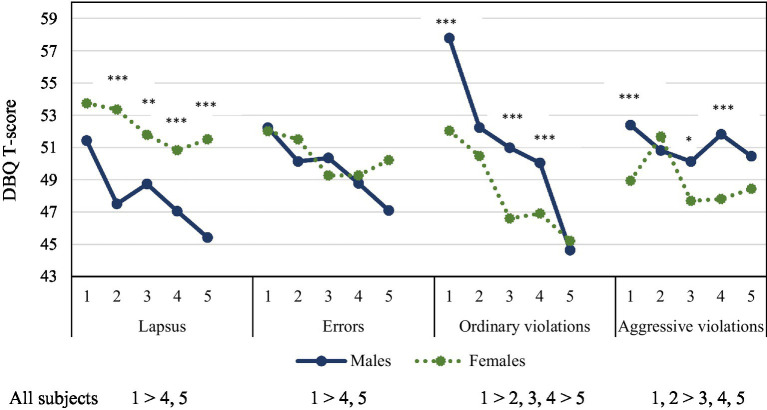
ANOVA mean comparison of age ranges and Driver Behavior Questionnaire (DBQ): 1: 30 years old and below; 2: 31–40; 3: 41–50; 4: 51–60, and 5: more than 60 years old. Scheffe post-tests comparisons (*p* < 0.05). *T*-test of gender comparisons: *p < 0.05, **p < 0.01, ***p < 0.001.

The differences between age in both genders in the four DBQ scales are presented in Figure 1. The youngest people, in both men and women, show higher scores in Lapses than older ones (1 > 4, 5). Women score significantly more than men in all age ranges. In errors, there were no differences between age ranges or genders. On the other hand, the youngest group clearly score higher in Ordinary violations than the other age groups, and men tend to score higher than women in 3 out of 5 groups. In aggressive violations, the youngest women (groups 1 and 2) scored higher than age groups 3, 4 and 5, and men also scored significantly higher than women in age groups 1, 3 and 4.

Complementary to this analysis, an ANOVA 2×5 (Gender x Age range) was performed to test the possible interaction effects between the gender and age variables. This analysis also allows for testing the effect size of the gender, as well as age variables and the interaction. For the Lapses scale, F was significant for gender (*p* < 0.001) and age (*p* < 0.001), but not for the interaction. For the error scale, it was only significant for age (*p* < 0.003). Regarding ordinary violations, there was a significant effect of gender (*p* < 0.001), age (*p* < 0.001) and the interaction (*p* < 0.04). This interaction effect is due to the higher mean score of the youngest men. Similarly, differences on Aggressive violations were associated with gender (*p* < 0.001), and age (*p* < 0.001). The interaction was also significant (*p* < 0.003). Again, this interaction effect is mainly due to the higher mean score of youngest men. However, it should be mentioned that the interaction effect, when significant, presented an eta squared (η2) of about 0.01 indicating a rather small effect on the DBQ scales. Age presented η2 higher than gender in all but the Lapses variable. The largest η2 obtained were for the age factor on Ordinary (0.10) and Aggressive violations (0.07). Both values suggest a medium effect size.

### 3.3. Inter-correlations analysis separately for gender, and empirical network partial correlations

Table 1 shows two intercorrelation matrices (one for each gender) between age and all the psychometric variables included in the present study. Correlations equal to or greater than 0.15 were statistically significant, but this result is due to the large size of the sample. We think it is more appropriate to focus only on correlations greater than 0.30. It should be remarked that the pattern of correlations was highly congruent across genders, although men tended to have higher correlations. Age was strongly and negatively related to Sensation Seeking for men and women (−0.37 and −0.36, respectively). Age was also negatively related to ordinary violations (−0.40 and −0.26), and aggressive violations (−0.34 and −0.17). As it has been stated, correlations tended to be lower for women. Extraverted people were less avoidant (−0.33 and −0.26). People with high scores on Neuroticism were more dependent (0.34 and 0.37), and avoidant (0.50 and 0.41), and people high on Sensitive to Punishment were also more Dependent (0.32 and 0.35), Avoidant (0.49 and 0.39) and Spontaneous decision-making (0.34 and 0.28) styles. Those more Sensitive to Punishment were more aggressive (0.38 and 0.30) and commit more violent violations while driving (0.36 and 28). Sensation seekers were more sensitive to reward (0.33 and 0.30) and tended to have a more spontaneous decision-making style (0.32 and 0.37) and more ordinary violations (0.28 and 0.30). The subjects with higher scores on Aggressiveness commit more aggressive driving violations (0.43 and 0.38) and have a more spontaneous decision-making style (0.36 and 0.35). People more Sensitive to Reward presented more aggressive driving violations as well (0.36 and 0.28).

**Figure 2:**
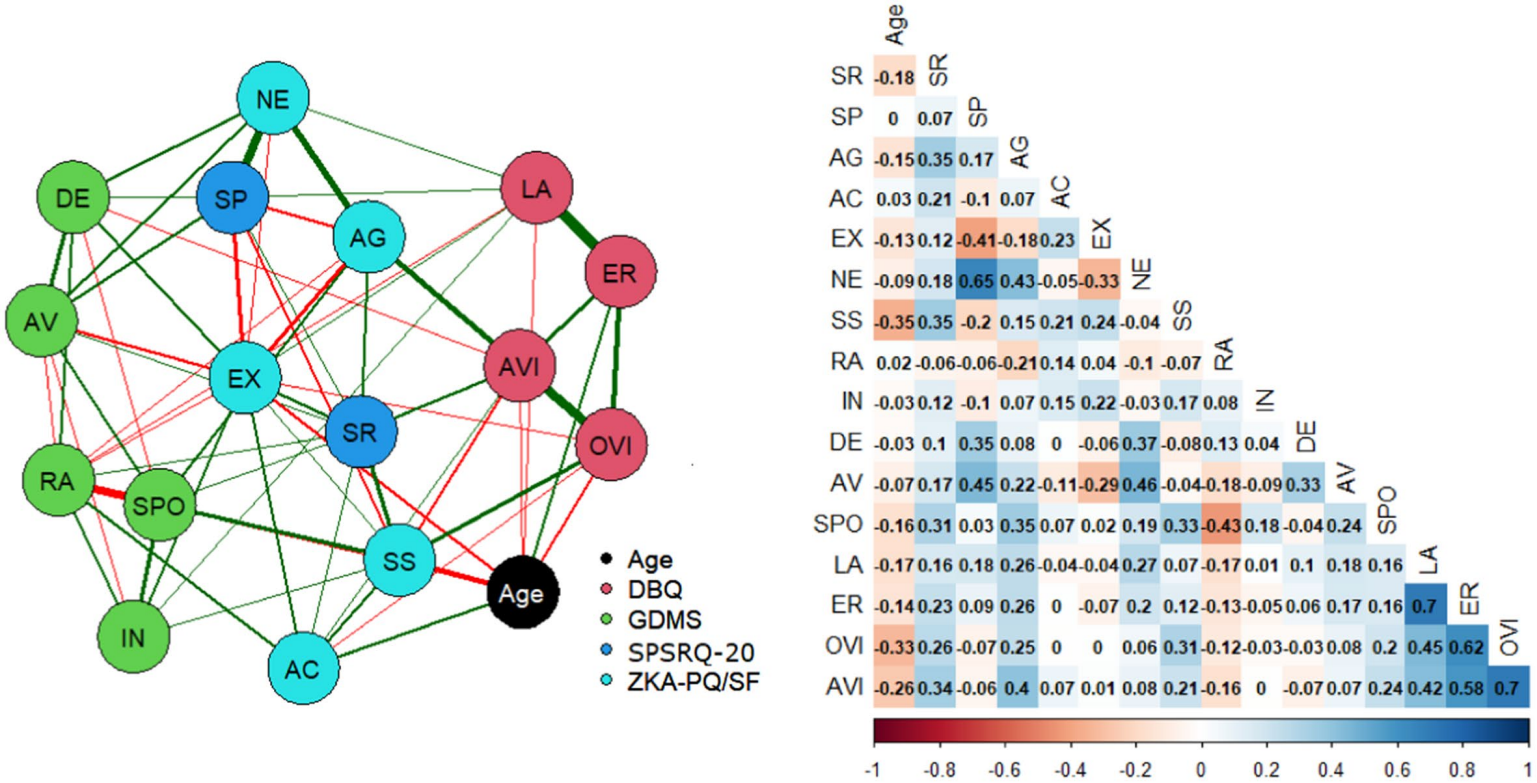
Empirical network with the age, DBQ, GDMS, SPSRQ-20, and ZKA-PQ/SF scales (partial correlations). Nodes represent scales. The edges represent the relationship among scales. The thicker the edge, the greater is the relationship between scales. Green and red lines represent positive and negative relationships, respectively. SR, Sensitivity to Reward; SP, Sensitivity to Punishment; AG, Aggressiveness; AC, Activity; Ex, Extraversion; NE, Neuroticism; SS, Sensation Seeking; RA, Rational; IN, Intuitive; DE, Dependent; AV, Avoidant; SPO, Spontaneous; LA, Lapses; ER, Errors; OVI, Ordinary violations; AVI, Aggressive violations.

As can be seen in Table 1, the zero-order correlations matrices have many variables that correlate with each other. In addition to the strong effect of age already mentioned, some personality variables also correlated strongly with certain decision-making scales. To eliminate the excessive effect of these correlations, a partial correlation was performed, controlling all variables. The use of partial correlations allowed a clearer interpretation of the real and direct relationships between scales. Figure 2 shows the empirical network as well as the partial correlations for the entire sample. Although correlations decreased in intensity, they retained the same pattern depicted above for the zero-order correlation matrices for men and women.

### 3.4. Factor convergence and regression analysis

Another way to study the relationships among risky driving scales, personality, and decision-making variables is to perform a Principal Components Analysis (PCA) with Varimax rotation (Table 3). Three factors were extracted according to Horn’s parallel analysis method (Horn, 1965). The Kaiser-Meyer-Olkin Measure of Sampling Adequacy (KMO) was 0.750, with an approximate Chi-Square of 5113.83 (*p* < 0.001). The variance accounted for by the three factors was 50.03%. The first factor grouped the four scales of DBQ and, with a lower loading, the Rational decision-making scale in negative. As the four DBQ scales present high and positive correlations and load on the same factor, it sounds psychometrically appropriate to compute a single score. This result allows us to compute a regression analysis on the DBQ single score what is more informative about the personality correlates of the risky driving behavior than using each DBQ separately. The second factor grouped the Neuroticism scales, including Sensitivity to Punishment, Extraversion (−), the decision-making scales related to Neuroticism (Avoidant and Dependent) and Aggressiveness. The third was a factor of Sensation Seeking, Sensitivity to Reward (as a measure of impulsivity according to Gray’s personality model) and Activity. The most disinhibited decision-making styles -Spontaneous and Intuitive- and Activity trait were also included. It is important to remark that Aggressiveness loaded on the three factors, and that the loading on its own factor is quite similar to the loading on the risky driving factor (the first).

**Table 3:** Principal component analysis including DBQ, ZKA-PQ/SF, SPSRQ-20, and GDMS scales.

	I	II	II
Errors	**0.85**	0.07	−0.06
Ordinary violations	**0.83**	−0.11	0.08
Aggressive violations	**0.82**	−0.07	0.16
Lapses	**0.73**	0.17	−0.07
Rational	**−0.29**	−0.16	−0.10
Neuroticism	0.16	**0.83**	0.04
Sensitivity to punishment	0.00	**0.81**	−0.18
Avoidant	0.15	**0.70**	0.01
Dependent	−0.09	**0.54**	0.03
Extraversion	−0.08	**−0.50**	**0.44**
Aggressiveness	**0.40**	**0.42**	**0.34**
Sensation seeking	0.23	−0.16	**0.65**
Sensitivity to reward	0.**30**	0.20	**0.61**
Spontaneous	0.27	0.24	**0.57**
Intuitive	−0.13	−0.05	**0.53**
Activity	−0.07	−0.11	**0.52**

Factor loadings equal to or greater than 0.30 are in boldface.

A multiple linear regression analysis was also carried out separately for men and women with the aim of identifying the best predictors of total DBQ score using age, personality traits and decision-making styles as independent variables (Table 4). Age (−), Aggressiveness (+), Sensitivity to Reward (+) and Rational decision-making style (−) explained 25% of risky driving (DBQ) in men. In women, the variables that entered the equation were age (−), Aggressiveness (+), Sensitivity to Reward (+), Rational decision-making styles (−) and Sensation Seeking (+), explaining 18% of the variance of risky driving (DBQ). The pattern of predictors is, therefore, highly congruent across genders.

**Table 4:** Standardized coefficients, *t*-test and significance of the variables included in the equation of the multiple regression analysis separately for genders, considering age, ZKA-PQ/SF, SPSRQ-20 and decision-making styles as independent variables and DBQ total score as dependent variable.

	Standardized coefficients	*t*	Sig.
Men. *R*^2^ adjusted: 0.25			
Age	−0.273	−6.996	0.001
Aggressiveness	0.216	5.207	0.001
Sensitivity to reward	0.170	4.102	0.001
Rational	−0.103	−2.659	0.008
Women *R*^2^ adjusted: 0.18			
Age	−0.122	−2.636	0.009
Aggressiveness	0.258	5.635	0.001
Sensitivity to reward	0.129	2.734	0.007
Rational	−0.116	−2.629	0.009
Sensation seeking	0.100	2.055	0.040

Table 5 shows the results of predicting every DBQ scale from age, personality scales and decision-making styles. It was observed that all variables accounted for between 11 and 29% of the variance of the DBQ scales. It should be remarked that aggressive violations were better predicted than lapses and errors. This better prediction came mainly from the Aggressiveness and Sensitivity to reward traits. Lapses and error presented a different pattern according to gender. For men, the best predictor was age, and for women Aggressiveness, Neuroticism (although significant for lapses only), and Sensation Seeking and Sensitivity to Reward (significant for errors only). Decision-making styles barely contributed to the variance, with a lack of stability in the pattern observed across DBQ scales.

**Table 5:** Standardized coefficients, beta,^a^ t and significance of the variables by gender included in the equation of the multiple regression analysis, considering age, ZKA-PQ/SF, SPSRQ-20, and decision-making styles as independent variables and DBQ domains as dependent variables.

Men	Lapses (*R*^2^ adj: 0.13)			Errors (*R*^2^ adj: 0.12)			Ordinary violations (*R*^2^ adj: 0.22)			Aggressive violations (*R*^2^ adj: 0.30)			β	T	Sig.	β	T	Sig.	β	t	Sig.	β	t	Sig.
Age	**−0.172**	−3.780	0.001	**−0.112**	−2.456	0.014	**−0.275**	−6.352	0.001	**−0.237**	−5.799	0.001
Aggressiveness	**0.126**	2.467	0.014	0.075	1.472	0.142	0.102	2.110	0.035	**0.322**	7.019	0.001
Activity	−0.056	−1.252	0.211	0.026	0.584	0.559	−0.055	−1.296	0.195	0.053	1.306	0.192
Extraversion	−0.016	−0.329	0.742	−0.061	−1.223	0.222	−0.057	−1.191	0.234	−0.033	−0.733	0.464
Neuroticism	0.080	1.297	0.195	0.106	1.712	0.087	0.048	0.820	0.412	−0.052	−0.938	0.348
Sensation seeking	−0.011	−0.234	0.815	−0.003	−0.064	0.949	0.130	2.786	0.006	−0.021	−0.484	0.628
Sensitivity to reward	0.087	1.761	0.079	**0.213**	4.320	0.001	**0.153**	3.278	0.001	**0.199**	4.500	0.001
Sensitivity to punishment	0.050	0.881	0.379	−0.024	−0.424	0.672	−0.084	−1.545	0.123	−0.071	−1.383	0.167
Rational	−0.088	−1.860	0.063	−0.065	−1.375	0.170	−0.026	−0.587	0.558	**−0.110**	−2.584	0.010
Intuitive	0.011	0.245	0.807	−0.088	−1.987	0.047	−0.063	−1.508	0.132	−0.032	−0.795	0.427
Dependent	0.040	0.852	0.395	−0.008	−0.180	0.858	−0.052	−1.187	0.236	−0.079	−1.898	0.058
Avoidant	0.067	1.281	0.201	0.089	1.692	0.091	0.017	0.344	0.731	0.025	0.539	0.590
Spontaneous	−0.052	−1.015	0.311	−0.090	−1.760	0.079	0.010	0.204	0.838	−0.006	−0.125	0.901
Women	Lapses (*R*^2^ adj: 0.10)			Errors (*R*^2^ adj: 0.11)			Ordinary violations (*R*^2^ adj: 0.16)			Aggressive violations (*R*^2^ adj: 0.19)			β	T	Sig.	β	t	Sig.	β	t	Sig.	β	t	Sig.
Age	−0.057	−1.150	0.251	−0.035	−0.714	0.475	**−0.183**	−3.799	0.001	**−0.118**	−2.483	0.013
Aggressiveness	**0.166**	3.061	0.002	**0.183**	3.394	0.001	**0.184**	3.512	0.001	**0.323**	6.251	0.001
Activity	−0.037	−0.765	0.445	−0.055	−1.161	0.246	−0.011	−0.243	0.808	0.010	0.215	0.830
Extraversion	0.069	1.266	0.206	−0.006	−0.103	0.918	−0.034	−0.646	0.518	0.029	0.565	0.572
Neuroticism	0.128	1.896	0.059	0.071	1.051	0.294	−0.037	−0.563	0.574	0.020	0.311	0.756
Sensation seeking	0.060	1.063	0.288	0.092	1.649	0.100	**0.162**	2.980	0.003	−0.010	−0.181	0.856
Sensitivity to reward	0.080	1.553	0.121	0.098	1.912	0.057	0.084	1.691	0.092	**0.169**	3.457	0.001
Sensitivity to punishment	0.025	0.384	0.701	−0.076	−1.154	0.249	−0.119	−1.869	0.062	−0.116	−1.848	0.065
Rational	**−0.114**	−2.108	0.036	−0.063	−1.164	0.245	−0.085	−1.630	0.104	−0.016	−0.303	0.762
Intuitive	−0.037	−0.760	0.448	−0.047	−0.976	0.330	−0.042	−0.902	0.368	−0.047	−1.018	0.309
Dependent	−0.001	−0.018	0.986	0.026	0.504	0.615	0.073	1.463	0.144	−0.033	−0.676	0.500
Avoidant	−0.004	−0.079	0.937	0.044	0.830	0.407	0.043	0.846	0.398	−0.019	−0.374	0.709
Spontaneous	0.017	0.290	0.772	0.062	1.043	0.297	0.001	0.022	0.983	0.054	0.949	0.343

^a^Significant (*p* < 0.05) beta coefficients are in boldface.

### 3.5. Non-parametric and multiple linear regression analysis

**Figure 3:**
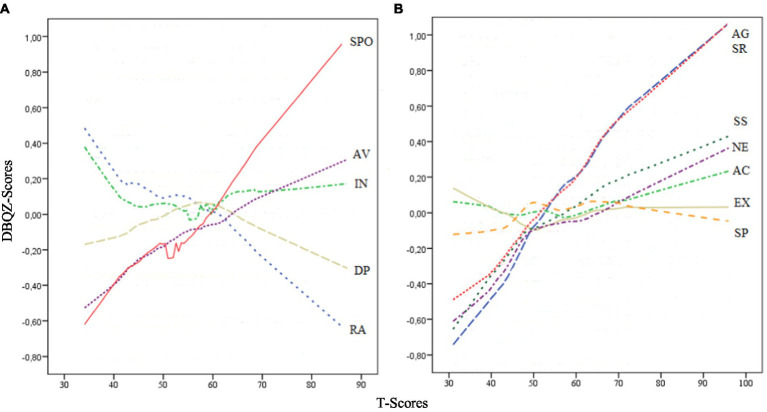
LOESS plots for GDMS **(A)**, and ZKA-PQ/SF and SPSRQ-20 **(B)**. RA, Rational; IN, Intuitive; DE, Dependent; AV, Avoidant; SPO, Spontaneous; AG, Aggressiveness; AC, Activity; Ex, Extraversion; NE, Neuroticism; SS, Sensation Seeking; SR, Sensitivity to Reward; SP, Sensitivity to Punishment.

Figure 3 shows a nonparametric LOESS graphical analysis for local regression for men and women together. Based on correlation and factor analysis results, total DBQ scores were used. They were converted into T-scores, and GDMS (A) and ZKA-PQ/SF and SPSRQ-20 into Z-scores (B). The mean of the T-score was 50 and the mean of the Z-scores was 0, therefore the domain lines of the GDMS and ZKA-PQ/SF and SPSRQ-20 coincided on the graph at these points. As subjects progressed in DBQ T-scores, the lines representing the domains of the GDMS, and the two personality questionnaires spread out on the graph. In the case of the GDMS scales, the most predictive were spontaneous (positive), and rational, (negative). In the case of ZKA-PQ and SPSRQ-20, Aggressiveness and Sensitivity to Reward presented the most positive prediction, followed by Sensation Seeking and Neuroticism. The rest presented flat lines, suggesting a lack of prediction of total DBQ score.

## 4. Discussion

This study analyzes altogether the role of age, gender, personality traits (Zuckerman’s and Gray’s models) and decision-making styles on risky driving in a large sample of middle social position and gender parity, and with an age distribution similar to that of the general population. Our purpose was to contribute to this field of research by deepening our knowledge about the role of individual difference variables such as personality traits and decision-making styles on motor vehicle driving behavior. First, this study replicates the gender differences on risky behavior scales. Women scored higher on lapses, and men on ordinary violations in all age ranges (Berdoulat et al., 2013; Lajunen et al., 2022). Men also scored higher on Aggressive violations than women, except for the group between 31 and 40 years old.

With regard to the role of independent variables, most studies indicate that age, and to a lesser extent gender, are strong predictors for risky driving behavior (McCartt et al., 2009; Lucidi et al., 2010; Perepjolkina and Renge, 2011; Barr et al., 2015; Zeyin et al., 2022). The results are in strong agreement with the literature, since age was also negatively associated with risky driving (McCartt et al., 2009; Zeyin et al., 2022). In fact, the results suggest that age is the best predictor of risky driving behavior in men.

More centrally to the aims of the study, personality traits proved to be associated with risky driving in both men and women. The personality trait most associated with risky driving was Aggressiveness in both genders. These results for Aggressiveness and driving are consistent with the findings of Iancu et al. (2016), but not entirely with those of Šeibokaitė et al. (2014), who found that Aggressiveness was a predictor of risky driving only in men. Along with Aggressiveness, Sensitivity to Reward (Impulsiveness) predicted risky behavior in both genders, and Sensation Seeking also seemed to play a role (Lucidi et al., 2019). Considering the theoretical relationship between Sensation Seeking and Sensitivity to Reward, results of the present study suggest that an approach behavior in response to incentives (Corr, 2004) could be responsible for some risky driving behaviors. These incentives could be from arriving earlier to some kind of competitive behavior. Considering the relationships among DBQ and personality scales, Neuroticism was also observed to be associated with committing more lapses, hence providing some confirmation that being prone to anxiety and worries leads to a larger amount of errors (especially in women) (Alavi et al., 2017; Wang et al., 2018). Sensitivity to Punishment played a less relevant role, and in women only. A possible explanation is that the criteria are focused on risky behavior, where the need to obtain something is clearly more important than any possible punishment resulting from negative outcomes of risky behavior.

A contribution of this study is the fact that decision-making styles add predictive power to risky driving beyond personality traits. Congruent with the literature, rational style is the decision-making style most related with risky driving behavior. Note that the relationship is negative, so this decision-making style seems to act as a protector in risky driving, in agreement with other studies (Chen, 2013). This result (albeit with a small sample size) suggests that an objective analysis of the situation could mediate the impact of the emotion (whether aimed at getting reward such as impulsiveness, fighting someone such as aggressiveness and anger, or feeling anxiety). On the other hand, Spontaneous style is the one most closely related to Ordinary and Aggressive violations in both genders. Avoidant style is also associated with Lapses and Errors, although to a lesser extent in women.

Our results corroborate the findings of previous research that has studied the relationships between personality and decision-making scales and risky driving separately. In our study, we have tried to investigate their relationships in a more integrated way. Furthermore, to the best of our knowledge, this is the second study to use the ZKA-PQ in driving behavior research. Note that most studies have used the FFM. In addition, the inclusion of a personality measure according to Gray’s model makes it possible to analyze the role of impulsivity (defined as Sensitivity to Reward) from a complementary perspective, more inspired by the biological tradition of personality research.

Considering the linear results observed in the present paper, drivers with high scores on risky driving could be defined as young, more aggressive, high in Neuroticism (and Sensitivity to Punishment), Sensation seekers, and low in rational, and high in avoidant and spontaneous decision-making styles. It prompts the following considerations: (1) It provides some insight into the phenomenon that the overall crash rate steadily decreases as driver age increases. There is ample evidence that age is strongly negatively associated with disinhibited personality traits such as Sensation Seeking, Aggressiveness, and Sensitivity to Reward (Zuckerman, 1994, 2005; Jonah, 1997; Jonah et al., 2001; Iancu et al., 2016; Scott-Parker and Weston, 2017; Zhang et al., 2019). In addition, the decision-making style most related with risky driving (Rational), and which acts as a protective factor, increases with age (Pellerone et al., 2016). The results of the present paper would suggest that changes in personality traits and decision-making styles could contribute to lower risk driving as age increases. (2) In Spain, we have a points-based driver’s license. Drivers who lose their driving license because they have run out of points are forced to take a driver awareness and re-education course to recover it. The present results suggest that this course should consider psychological aspects to increase its efficacy and reduce the likelihood of traffic accidents in the future, especially in the case of young people. In this sense, courses should include psychosocial interventions that have been successful to reduce aggressiveness and aggressive behaviors (McGuire, 2008). In addition, interventions aimed to promote self-control (that is to say, less impulsive behavior) would also be advisable. This intervention would help to develop self-regulatory skills that would inhibit risky behavior (De Ridder et al., 2020).

As a secondary aim of the present study, we replicated the observed relationships among variables analyzed in the present study. As has been commented above, the negative relationship among age and some personality traits was replicated. Gender differences in personality traits match those expected according to the literature. Women score higher on Neuroticism and Sensitivity to Punishment, and men on Sensation seeking and Sensitivity to Reward (Aluja and Blanch, 2011; Cross et al., 2011, 2013; Rossier et al., 2016). There were no gender differences in Aggressiveness. This is due to the new conceptualization of Zuckerman’s trait in the ZKA-PQ (Aluja et al., 2010). It includes four facets (Physical aggression, Verbal Aggression, Anger, and Hostility) with different patterns on gender differences (Aluja et al., 2018; Björkqvist, 2018). With regard to decision-making styles, and in line with the findings of Delaney et al. (2015) and Bayram and Aydemir (2017), women had higher scores on the Dependent and Intuitive styles.

This study has strengths and limitations. Among the strengths is the large sample of anonymous drivers with gender parity and a wide age range, the use of questionnaires validated in the same sociocultural context, and the good internal consistency of the different scales. The basic limitation is the fact that the study was carried out with data obtained through self-reported questionnaires. Cross-sectional surveys have an inherent method bias. Independent and dependent variables are measured at the same time, which makes it difficult to distinguish between cause and effect. In this sense, it would be desirable to use longitudinal designs using, for instance, a panel design. Thus, both individual and situational factors for risky driving could be better detected. It would also be advisable to use objective data on risky driving such as loss of points, number of sanctions or accidents, experience as a driver, and so forth, to complement self-report measures. For instance, not considering the exposure of the driver to risk, the frequency of driving or the usual mileage of the participant as covariates is a limitation of the present study (Lucidi et al., 2010). These variables could be a key confounder for the effect of age, gender, personality traits and decision-making styles. Another limitation is that the sample is composed of habitual drivers of motor of 2 or 4-wheel vehicles, but we have not recorded the normal vehicle used. People who ride motorcycles could present a larger number of risky driving behaviors (Peek-Asa and Kraus, 1996; Hassanzadeh et al., 2020). A future study should compare both groups of drivers of 2 or 4-wheel vehicles to test if relationships observed remain invariable across vehicle type.

Summing up, the psychological description of risky drivers obtained in our large and representative sample of drivers is mainly characterized by young subjects, high scores in Aggressiveness, Neuroticism, Sensation Seeking, Sensitivity to Reward and Punishment, and decision-making styles characterized by low rationality, and high spontaneity. The present study demonstrates that personality traits and decision-making styles play a complementary role in the prediction of risky behavior. Finally, the present study closely replicates in the Spanish population the association of age with risky driving, as well as the different pattern of risky behaviors observed in men and women in other countries.

## Data availability statement

The datasets presented in this article are not readily available because they are subject to ongoing research. Requests to access the datasets should be directed to the corresponding author.

## Ethics statement

The studies involving human participants were reviewed and approved by the Ethical Commission of the University of Lleida. The patients/participants provided their written informed consent to participate in this study.

## Author contributions

AA and FB designed this study and conducted the statistical analyses. All authors collected the data, contributed to the writing and editing of the manuscript, and approved the final version.

## Funding

This research was funded by a grant from the Spanish Ministry of Economy, Industry and Competitiveness (PID2019-103981RB-I00). All the authors took part in this project as researchers.

## Conflict of interest

The authors declare that the research was conducted in the absence of any commercial or financial relationships that could be construed as a potential conflict of interest.

## Publisher’s note

All claims expressed in this article are solely those of the authors and do not necessarily represent those of their affiliated organizations, or those of the publisher, the editors and the reviewers. Any product that may be evaluated in this article, or claim that may be made by its manufacturer, is not guaranteed or endorsed by the publisher.

## Footnotes

^1^ https://revista.dgt.es/es/noticias/nacional/2022/01ENERO/0107-Balance-prov-accidentalidad-2021.shtml
